# Potential of Personalized Dendritic Cell-Based Immunohybridoma Vaccines to Treat Prostate Cancer

**DOI:** 10.3390/life13071498

**Published:** 2023-07-01

**Authors:** Simon Hawlina, Robert Zorec, Helena H. Chowdhury

**Affiliations:** 1 Clinical Department of Urology, University Medical Centre Ljubljana, 1000 Ljubljana, Slovenia; 2 Department of Surgery, Faculty of Medicine, University of Ljubljana, 1000 Ljubljana, Slovenia; 3 Laboratory of Cell Engineering, Celica Biomedical, 1000 Ljubljana, Slovenia; 4 Laboratory of Neuroendocrinology–Molecular Cell Physiology, Institute of Pathophysiology, Faculty of Medicine, University of Ljubljana, 1000 Ljubljana, Slovenia

**Keywords:** prostate cancer, immunotherapy, dendritic cell-based vaccines, castration-resistant prostate cancer, tumor microenvironment, biomarkers

## Abstract

Prostate cancer (PCa) is the most commonly diagnosed cancer and the second most common cause of death due to cancer. About 30% of patients with PCa who have been castrated develop a castration-resistant form of the disease (CRPC), which is incurable. In the last decade, new treatments that control the disease have emerged, slowing progression and spread and prolonging survival while maintaining the quality of life. These include immunotherapies; however, we do not yet know the optimal combination and sequence of these therapies with the standard ones. All therapies are not always suitable for every patient due to co-morbidities or adverse effects of therapies or both, so there is an urgent need for further work on new therapeutic options. Advances in cancer immunotherapy with an immune checkpoint inhibition mechanism (e.g., ipilimumab, an anti-CTLA-4 inhibitor) have not shown a survival benefit in patients with CRPC. Other immunological approaches have also not given clear results, which has indirectly prevented breakthrough for this type of therapeutic strategy into clinical use. Currently, the only approved form of immunotherapy for patients with CRPC is a cell-based medicine, but it is only available to patients in some parts of the world. Based on what was gained from recently completed clinical research on immunotherapy with dendritic cell-based immunohybridomas, the aHyC dendritic cell vaccine for patients with CRPC, we highlight the current status and possible alternatives that should be considered in the future.

## 1. Introduction

Prostate cancer (PCa) is the most commonly diagnosed form of cancer in men and the second most common cause of cancer-related death in the developed world [1]. About a third of patients with PCa who have been castrated develop a castration-resistant form of PCa (CRPC), which is currently incurable. Cancer cells in CRPC are no longer sensitive to androgen deprivation, which is the basic form of treatment for the advanced stage of the disease, therefore additional therapies are usually considered, including chemotherapy, radiation therapy, second-generation antiandrogens and immunotherapy [2]. Treatment prolongs survival and preserves quality of life. However, the optimal combination and sequence of these therapies is not yet known. Not all therapies are always suitable for every patient due to co-morbidities or adverse effects of therapies or both; therefore, CRPC represents an unmet medical need.

In recent years, immunotherapy has greatly influenced the treatment of metastatic cancer and changed the standard of care for many types of tumors. Immunotherapeutic options in the fight against malignancies are numerous and can be used in different ways. In the development of carcinogenesis, cancer cells escape the control of the immune system. Thus, the main goal of immune therapies is to regain control by triggering specific immune reactions similar to those that occur during spontaneous tumor rejection. Current immunotherapy strategies include monoclonal antibodies against molecules that regulate the immune response (immune checkpoint inhibitors (ICIs)), cell therapies such as the transfer of ex vivo-activated T cells and natural killer (NK) cells, and cancer treatment vaccines, which use tumor antigens (TAs) to activate the patient’s immune system against cancer cells [3,4,5,6]. Although some disseminated cancers, such as malignant melanoma, lung cancer and renal cell carcinoma, have shown robust responses to immunotherapy with monoclonal ICIs, prostate cancer has generally not shown a significant response [7]. Advances in cancer immunotherapy with an immune checkpoint inhibition mechanism (e.g., ipilimumab, an anti-CTLA-4 inhibitor) have not shown a survival benefit for patients with CRPC [8,9]. With the exception of cell-based therapy with sipuleucel-T [10] and immunotherapy with dendritic cell-based immunohybridomas, the aHyC dendritic cell vaccine [11,12], other immunological approaches have not given clear results and have indirectly prevented the breakthrough of this type of therapeutic strategy into clinical use [13,14,15].

The heterogeneity of PCa, resistance to treatment and increasing need for personalized therapies are driving the latest research to combine different treatment approaches and introduce new ones. Many new therapies are already based on modulation of the immune system with the aim of enhancing the “visibility” of the tumor antigen to the patient’s immune system and/or interrupting various tumor survival strategies. In addition, standard oncologic therapies (e.g., chemotherapy, radiotherapy, androgen deprivation therapy) not only act as cytotoxic agents for PCa tumor cells but also have an immunological effect, because cell lysis causes a release of TAs and possibly affects the activation of cytotoxic T cells [16,17], i.e., a mechanism is triggered similar to that with cancer treatment vaccines.

In this article, we summarize the current status, the progress in development, possible limitations and future directions of a pleotropic antigen-targeting immunotherapy for PCa, particularly cell-based immunotherapy, considering the new insights arising from our recent study [11].

## 2. Cancer Treatment Vaccines

Most cancer treatment vaccines are based on the use of TAs, which can be tumor-associated antigens (TAAs) or more rarely, tumor-specific antigens (TSA), to activate the patient’s immune system through cascade-regulating diverse immune cell activity, starting with the entry of the TAA into antigen-presenting cells (APCs), which then present the antigen together with the molecules of the major histocompatibility complex (MHC) to naive lymphocytes. Several types of lymphocytes are activated in this process, including CD4^+^ and CD8^+^ cells, which theoretically could lead to both specific cellular immunity and humoral immune responses against tumor cells, promoting their destruction and preventing tumor growth [18]. Thus, cancer treatment vaccines are generally composed of an adjuvant that functions to activate APCs and a target protein or peptide known to be associated with the cancer [7]. After intravenous, subcutaneous or intradermal injection, antigen-loaded APCs, usually dendritic cells (DCs), migrate to the draining lymph nodes where they present small peptide fragments of the target antigen on MHC molecules to prime T cell recognition.

Historically, the first cancer treatment vaccine based on tumor cells and tumor lysates was developed in 1980. Scientists used autologous tumor cells to treat colorectal cancer [19]. The first human TSA was identified in melanoma in the early 1990s [20]. This opened a new chapter in the use of TAs in cancer vaccines. In 2010, a cell-based treatment vaccine (sipuleucel-T) was successfully used to treat PCa [10]. In 2011, the Nobel Prize in physiology or medicine was awarded for discovering the role of DCs in the immune system. The scientific editors selected the published research of scientist Topalian and colleagues in the field of cancer immunotherapy as the breakthrough article of 2013 [21]. In 2018, the Nobel Prize was awarded to James Allison (University of Texas MD Anderson Cancer Center) and Tasuku Honjo (Kyoto University School of Medicine) for their discoveries leading to new approaches in harnessing the immune system to fight cancer, consisting of checkpoint inhibition mechanisms. The recent outbreak of the coronavirus pandemic has spurred the development of vaccine technology and put cancer vaccines back in the spotlight. Currently, many cancer vaccines are still in the preclinical and clinical research stages [22].

Cancer treatment vaccines can be broadly categorized into four different types based on the way TAs are introduced and presented to the immune system: nucleic acid-, peptide-, viral vector- and cell-based vaccines, as described in the following sections for the treatment of PCa and in Table 1.

### 2.1. Nucleic Acid-Based Vaccines

Nucleic acid vaccines contain DNA or RNA encoding TA. RNA-based vaccines consist of TA-encoding mRNA. The use of mRNA as a cancer treatment vaccine has several advantages: a high level of safety due to the impossibility of incorporation into the genome, i.e., without insertional mutagenesis and the absence of the introduction of an infectious virus; enables the simultaneous delivery of several antigens for different TAs; the RNA only needs to be internalized into the cytoplasm, which is immediately followed by antigen(s) expression; can elicit humoral and cell-mediated immune responses, thereby increasing the likelihood of overcoming vaccine resistance; and efficient manufacturing. On the other hand, there are also challenges in using mRNA, such as a short half-life and only transient protein expression. Currently, several mRNA cancer vaccines are in clinical trials for various types of cancer [23] (melanoma, lymphoma, colorectal cancer); however, only one RNA-based vaccine has been tested for the treatment of prostate cancer, which failed to show a survival benefit despite increased immunogenicity [24,25]. Nevertheless, combination therapy with an mRNA vaccine and immune checkpoint inhibitors shows better prospects for cancer treatment [26], and a similar combination therapy for prostate cancer may prove beneficial in the future.

DNA-based vaccines consist of genetically modified DNA, usually in the form of plasmids that contain the coding sequence of the target antigen. They can be delivered by a variety of routes as well as by different strategies (e.g., electroporation, sonoporation, gene gun). The antigen encoded by the DNA vaccine is then expressed and presented on the MHC molecules for T cell activation. An advantage of DNA vaccines is the activation of both innate and adaptive immunity [27,28,29]. Another important advantage is that they promote a systemic immune response and immunological memory [30]. However, DNA vaccines typically exhibit relatively poor immunogenicity, especially in clinical trials, mostly due to poor DNA uptake into cells and due to the various mechanisms of resistance during tumor development [31,32].

DNA-based vaccines containing information on various TAAs, such as the prostate-specific antigen (PSA), prostate-specific membrane antigen (PSMA), prostatic acid phosphatase (PAP), androgen receptor (AR) and testicular cancer antigen, have not demonstrated increased clinical efficacy but most trials have shown an immunological response [33]. In addition to shared TAAs, such as AR and PAP, the DNA vaccine platform can generate personalized cancer vaccines for patients with PCa [34]. An ongoing phase 1 clinical trial (NCT03532217) utilizes a combination of a neoantigen DNA vaccination, nivolumab, ipilimumab and PROSTVAC for patients with metastatic hormone-sensitive prostate cancer (mHSPC), which takes advantage of both shared and personalized antigen approaches.

### 2.2. Peptide-Based Vaccines

Peptide-based vaccines are built of subunits containing the specific epitope of the tumor antigen [35]. After intradermal injection, professional APCs in the skin are exposed to the synthetic vaccine peptides, corresponding products and antigens associated with cancer. In a phase 1/2 clinical trial (NCT01784913) for patients with metastatic hormone-sensitive prostate cancer (mHSPC), UV1, a synthetic long-peptide vaccine containing fragments of human telomerase reverse transcriptase (hTERT) was administered in combination with granulocyte–macrophage colony-stimulating factor (GM-CSF). hTERT is normally repressed in healthy cells, but is usually overexpressed in cancer cells, and is responsible for the immortality of tumor cells [36]. Another rapidly evolving approach is the development of personalized peptide vaccines that involve identifying peptide candidates for individual patients for their ability to induce an immune response in vitro and of subsequent administration to the patient [37].

### 2.3. Viral Vector-Based Vaccines

These vaccines consist of viruses as a vector to transfer the gene-encoding TA(s) into patients, resulting in stimulation of the host’s immune response against the antigen [38,39]. In 2003, a phase 2 clinical study for patients with minimally symptomatic CRPC was conducted with PSA-TRICOM (PROSTVAC-VF), a virus-based vaccine using a combination of two viral vectors. Each vector encodes for PSA and three immune costimulatory molecules [40,41]. The virus infects APCs and this triggers cell surface protein expression and subsequent interaction with T cells, which in turn enhances the targeted immune response and cell-mediated destruction of tumor cells [42,43]. The PROSTVAC-VF vaccine was well tolerated. Overall survival was prolonged compared with the control group (25.1 months versus 16.6 months). However, the primary objective, which was to increase the time to progression, was not achieved. Patients with a higher disease burden had less benefit [42,44]. PSA-TRICOM did not receive US Food and Drug Administration (FDA) approval based on the findings of these trials.

### 2.4. Cell-Based Vaccines

The source of TAs to be introduced to the immune system can also be whole cells, autologous or allogeneic [45]. These are usually utilized in combination with GM-CSF to induce the growth and differentiation of DCs involved in antigen presentation [46].

#### 2.4.1. Tumor Cell-Based Vaccines

In this approach, the whole tumor cell is used as an antigen, which in turn facilitates both humoral and cellular immune responses. Tumor cells can be autologous or allogeneic and are usually genetically modified to express the immune stimulatory cytokine GM-CSF. GM-CSF induces the recruitment of APCs, which initiates a cascade of immune responses [47]. GVAX is a whole tumor cell-based vaccine against PCa and is genetically modified to secrete GM-CSF and irradiated to prevent further cell division. Although phase 1 and 2 studies confirmed clinical efficacy and safety, two phase 3 trials, VITAL-1 and VITAL-2, failed to show a clinical benefit [47,48,49]. There are attempts to improve the efficacy of GVAX-PCa by combining it with ICIs [50].

#### 2.4.2. Dendritic Cell-Based Vaccines

Today, the only approved modality of immunotherapy for patients with CRPC is cell-based medicine using the power of DCs. In the United States, it is available as sipuleucel-T [10] and in Slovenia (EU) as the recent next-generation medicine (Box 1), aHyC (autologous hybridoma cells), which consists of DCs electrofused with autologous tumor cells [11]. Although both medicines are based on autologous cells, they differ significantly in terms of administration and other properties.

Box 1 **A note on the regulatory frame regarding personalized medicine.**

The preparation of the innovative vaccine aHyC is available for international patients and approved in Slovenia by the Agency for Medicinal Products and Medical Devices of the Republic of Slovenia (part of the European Agency of Medicines—EMA). aHyC is a cell-based advanced therapy medicinal product (ATMP), for which the regulatory framework was established relatively recently, in 2009, in the EU. aHyC is completely personalized in nature; hence, this contributes to the relatively very high safety profile. Moreover, the number of patients enrolled in the clinical trial was relatively small, as it is for many other ATMPs [51], predominantly due to the nature of targeted diseases including orphan disease indications, unmet needs and pediatric patient populations, yet the trial was controlled and randomized, which is the standard for generating evidence in terms of efficiency and safety, but is a challenge for most ATMPs [52].

The most efficient, often designated “professional” APCs in the body are DCs. They play a key role in the activation and regulation of the acquired immune response; thus, they have been used extensively for the preparation of antitumor vaccines. After recognizing and binding, generally foreign antigens, they present them to other effector immune cells and thereby initiate a cellular immune response cascade. DCs are able to activate both naive and memory T lymphocytes and are thus the most suitable cell entity for amplifying the antitumor immune response [53]. Antigenic tumor material can also be provided to the patient’s immune system by equipping DCs with TAs. This is achieved by incubating DCs with tumor apoptotic bodies, with tumor necrotic lysates or with proteins, peptides or even mRNA alone. Such vaccines are prepared from the patient’s own immune cells, which can be exposed ex vivo to TAs and then introduced back into the patient, where they are supposed to boost the immune response to cancer cells in the body [54,55]. DCs have been used in clinical trials as a form of therapeutic treatment in cancer patients, including PCa for more than three decades, demonstrating that such an approach is safe (usually with only a few non-serious side effects), can trigger antitumor immunity and, in some cases, can prolong survival. However, the clinical efficacy (e.g., time to disease progression, symptoms) has been shown to be modest, although an immune response has been demonstrated in many cases (reviewed by Sutherland et al. [56]). One such example is sipuleucel-T, an autologous cell vaccine generated from a patient’s white blood cells, containing around 20% of DC markers, activated with a recombinant fusion protein (PA2024) to which a TSA (PAP) has been added. PAP is a glycoprotein enzyme synthesized by prostate epithelial cells, and its expression significantly increases in the progression of PCa [57,58]. The patient’s white blood cells are incubated ex vivo with the recombinant protein PA2024 consisting of PAP fused to GM-CSF, allowing the APCs to present the antigen on their surface [59]. The cell suspension is then re-infused intravenously into the patient (50 × 10^6^ CD54^+^ cells/250 mL suspension). Based on the results of the multicenter IMPACT study, the FDA approved sipuleucel-T in 2010 for the treatment of patients with CRPC with minimal or no symptoms. They reported a 4.1-month increase in survival compared with the placebo (25.8 versus 21.7 months [10]).

DCVAC is another known example of a DC-based vaccine. It is composed of activated DCs and dead cells of the prostate cancer cell line, LNCaP. Phase 1 and 2 studies showed improved survival in patients who received docetaxel and the DCVAC vaccine in combination [60]. However, no improvement in survival was found in a phase 3 study [61].

The DC immunotherapy strategy can be improved by a completely personalized approach. One effective way to achieve exposure of TAs is fusion of the plasma membranes of DCs and tumor cells of the same patient; the resulting hybrid cells, so-called immunohybridomas, mediate functions of original cells. They have the properties of APCs and contain both known and unknown TAs, derived from tumor cells. Recently, one such completely autologous DC-based cell vaccine has been tested in a phase 1/2 randomized, placebo-controlled trial by preparing DCs from the patient’s monocytes and using the electrofusion method to merge their plasma membranes with the patient’s own (autologous) cancer cells into immunohybridomas, termed aHyC, that were administered subcutaneously to the patients enrolled in the study [11,12]. The results revealed that the median overall survival was 58.5 months (95% confidence interval [CI], 38.8–78.2 months), and was inversely correlated to the subpopulation of NK cells in the peripheral blood, which were attenuated with the aHyC application, demonstrating modulation of the patient’s immune response [11]. Median cancer-specific survival was prolonged by 33 months compared with controls, almost 7 years after the diagnosis of CRPC [12].

Electrofusion of tumor cells and DCs to form hybridomas has been previously developed and evaluated with confocal microscopy and flow cytometry [62,63]. Antigen presentation also involves late endocytotic compartments (lysosomes) containing MHC II molecules, so heterologous fusion of vesicles (from different cell types, heterologous) is required to deliver antigens to MHC II molecules in hybridomas. It has been shown that fusion of late endocytotic compartments also occurs in aHyC hybridomas and that the efficacy of this approach, measured as an increased in vitro cytotoxic T cell response, is stronger when the proportion of fused late endocytic compartments is greater in electrofused hybridoma cells [64,65]. Furthermore, the advantage of the fusion of autologous tumor cells and DCs over other forms of vaccines is that such immunotherapy is not limited to only those types of tumors in which the potentially immunogenic antigenic determinants are currently well known. They are also effective against unidentified TAs, which arise from fused tumor cells and are bound to MHC class I and II molecules by specialized antigen presentation mechanisms of DCs, and presented to T lymphocytes for recognition [66].

In addition, in all forms of DC-based vaccines, DCs also express essential costimulatory molecules for effective activation of T lymphocytes and produce pro-inflammatory cytokines (e.g., interleukin-12). Thus, they can activate antigen-specific antitumor CD4^+^ and CD8^+^ clones of T lymphocytes in a balanced manner [64,67]. The activation of CD4^+^ T cells is necessary for long-term stimulation of the formation and functioning of antitumor effector CD8^+^ T lymphocytes, which ultimately destroy cancer cells and reduce the tumor burden. Research into the production and use of cell hybridomas has progressed all the way to clinical trials. It has been shown that patients with various diffuse forms of cancer tolerate this type of treatment well, with effector immune antitumor mechanisms proven to be reactivated, but objective tumor regression was confirmed only in a small number of patients [54,68]. Nevertheless, in vitro studies unequivocally demonstrate a significantly stronger activation of T lymphocytes with aHyC than with any other DC-based vaccines [3,69]. Therefore, the future of treatment with hybridomas obtained from tumor cells and DCs, such as aHyC, is very promising among cell-based vaccines.

**Table 1:** Clinical trials conducted with cancer treatment vaccines.

**Nucleic Acid-Based Vaccines**						
**Coding TA Epitope**	**Vaccine or Plasmid Vector**	**Adjuvants**	**Target; Phase**	***n*** **, Appl. Route**	**Outcomes**	**Ref/** **Year**
RNA-based						
Full-length STEAP1 PSA, PSMA, PSCA	CV9103	/	advanced CRPC; Phase I/IIa 2008-003967-37	44, i.d.	The RNA vaccine CV9103 was well tolerated and immunogenic. A total of 26 of 33 evaluable patients treated at the recommended dose developed an immune response to one or more antigens.	[24] /2015
Full-length STEAP1 PSA, PSMA, PSCA, PAP and MUC1	CV9104	/	advanced CRPC; Phase I/IIb 2011-006314-14	134, i.d.	CV9104 did not improve OS compared to placebo. No significant differences in the rPFS endpoints and time to symptom progression compared to placebo.	[25]/ 2017
DNA-based						
PSA	pVAX/PSA	GM-CSF, IL-2	CRPC; Phase I	9	In 25% cases (2), a PSA-specific cellular immune response and a rise in anti-PSA IgG. No AE (WHO grade > 2).	[70]/ 2004
			CRPC; Phase I	6	Induction of PSA-specific cellular immune responses in some cases.	[71]/ 2005
Full-length PAP	pTVG-HP [MVI-816]	GM-CSF	stage D0 PCa; Phase I/IIa	22, i.d.	No significant AE. PAP-specific CD4+ and/or CD8+ T cell proliferation (41% of patients); PAP-specific IFN gamma-secreting CD8+ T cells (14%).	[72,73]/2009 2010
			CSPC; Phase II NCT01341652	99, i.d.	Vaccination had detectable effects on micrometastatic bone disease.	[74]/ 2019
Modified PSA (rhesus PSA)	pVAXrcPSAv531	/	PCa with BCR; Phase I NCT00859729	15, i.d., EP	No systemic toxicity. Specific T cell reactivity PSA was observed in some patients.	[75]/ 2013
AR LBD (androgen receptor ligand-binding domain)	pTVG-AR	±GM-CSF	mCSPC; Phase I multicenter	40	Delayed the time to castration resistance; 28% had a PSA progression event. No grade ≥ 3 AE. In total, 47% developed Th1-type immunity to the AR LBD with a significantly prolonged PPFS vs. patients without immunity.	[76]/ 2020
**Viral Vector-Based Vaccines**						
**Coding TA Epitope**	**Virus Vector**	**Adjuvants**	**Target; Phase**	***n*** **, Appl. Route**	**Outcomes**	**Ref/** **Year**
PSA	rV-PSA	±GM-CSF	PCa after radical prostatectomy or radiation therapy; Phase I	33	Safe. Specific T cell response to PSA-3. In 42% of cases, stable disease for 6 months, in 27%, for 11–25 months.	[77]/ 2000
			advanced mPCa; Phase I	42, d.s., s.c.	No significant treatment-related toxicity; increase in the proportion of PSA-specific T cells after vaccination in some patients.	[78]/ 2002
PSA	rF-PSA/rV-PSA	/	advanced PCa; Phase II	64	Minimal toxicity; increase in PSA-specific T cell responses; free of PSA and clinical progression after 19 months.	[79]/ 2004
MUC-1	VV/MUC-1/IL-2 (vaccinia virus expressing MUC-1 and IL-2)	IL-2	advanced PCa; Phase I	16, i.m.	Safe and well tolerated. MHC-independent MUC-1-specific cytotoxic T cell activity; 1 patient had an objective tumor response.	[80]/ 2004
5T4 (trophoblast glycoprotein) (TroVax)	Vaccinia Ankara Virus	±GM-CSF	mCRPC; Phase II	27, i.m.	Safe and well tolerated. 5T4-specific antibody responses, robust 5T4-specific immune responses correlated with time to progression, no objective clinical responses.	[81]/ 2008
PSA	adenovirus/PSA	/	metastatic PCa; Phase I	32 s.c.	Safe with no serious AE. In total, 34% of patients produced anti-PSA antibodies, 68% produced anti-PSA T cell responses, PSA-DT was increased in 48%.	[82]/ 2009
PSA	rV-PSA/rF-PSA	GM-CSF	mCRPC; Phase II	32	Enhanced mOS. PSA-specific T cell responses showed a trend (*p* = 0.055) toward enhanced survival.	[44]/ 2010
PSA	PROSTVAC-VF (rV-PSA/rF-PSA)	+GM-CSF + 3 costimulatory molecules	mCRPC; Phase II	82/125	Longer mOS by 8.5 months (25.1 vs. 16.6 months for controls)	[42]/ 2010
			locally recurrent or progressive PCa; Phase I	21, s.c., i.t.	Safe and feasible. Stable (10) or improved (9) PSA values. Improved serum PSA kinetics and intense post-vaccination inflammatory infiltrates were seen in the majority of patients.	[83]/ 2013
PSMA	PSMA-VRP (Venezuelan Equine Encephalitis virus)	/	mCRPC; Phase I	12	Safe; no toxicities were observed. No PSMA-specific cellular responses—dosing was suboptimal; few patients had a humoral response to PSMA.	[84]/ 2013
/	HVJ-E (inactivated hemagglutinating virus of Japan envelope)	/	CRPC; Phase I/II UMIN000006142	6 i.t. and s.c.	PSA response rate was 16.6% (1/6), NK cell activity was elevated, IL-6, IFN-α, IFN-β and IFN-γ levels were not affected.	[85]/ 2017
PSA	PROSTVAC-VF	± GM-CSF	mCRPC; Phase III	864	Safe, well tolerated, it had no effect on OS or AWE (alive without events).	[86]/ 2019
5T4 (trophoblast glycoprotein) (TroVax)	ChAd (chimpanzee adenovirus) and MVA (Modified Vaccinia Ankara)		early-stage PCa or stable disease; Phase I NCT02390063	40, i.m.	Excellent safety profile. 5T4-specific T cell responses detected in the majority of patients.	[87]/ 2020
PSA, brachyury and MUC-1	adenovirus 5 (Ad5)	/	mCRPC; Phase I NCT03481816	18	Tolerable and safe; no grade >3 treatment-related AE toxicities. In total, 100% of 17 patients mounted T cell response to at least one TAA; 47% of patients mounted immune responses to all three TAAs.	[88]/ 2021
**Peptide-Based Vaccines**						
**TA**	**Peptide**	**Stimulatory Adjuvants**	**Target; Phase**	***n*** **, Appl. Route**	**Outcomes**	**Ref/** **Year**
Complex carbohydrate hexasaccharide molecule	globo H +KLH	QS-21 immunological saponin	PCa patients; Phase I	20 s.c.	High-titer IgM antibodies against globo H; decline of the slope of the log of PSA concentration vs. time.	[89]/ 1999
SART1, SART2, SRAT3, p56^lck^, ART-1, ART-4, CypB	PPV (up to 5 selected peptides)	/	CRPC; Phase I	10, i.d.	Safe and well tolerated with no major AE. Increased CTL response to both peptides and cancer cells was observed in four (40%) patients. Anti-peptide IgG antibodies were also detected in post-vaccination sera of seven (70%) patients. Decrease in PSA level in some patients.	[90]/ 2003
HER-2/neu	E75	GM-CSF	advanced PCa; Phase I	17	Safe with only minor toxicities observed. Effective in eliciting an HER-2/neu-specific immune response.	[91]/ 2005
Thomsen–Friedenreich antigen	TF-KLH	QS21 immunological saponin	biochemically relapsed PCa; Phase I	20	All patients developed maximum IgM and IgG antibody titers by week 9; change in post-treatment logPSA slopes vs. pretreatment was observed.	[92]/ 2005
SART1, SART2, SART3, Lck, ART1, PAP, PSA PSMA, MRP	PPV (up to 4 selected peptides)	/	localized PCa; Phase I	10	Increased CTL response and the anti-peptide IgG titers were observed in the post-vaccination samples in 8 of 10. Number of infiltrating memory CD4 T (CD45RO+) cells was significantly larger in the vaccination group vs. control group. CD8(+) T cell infiltration was seen only in the vaccinated group.	[93]/ 2007
PSA	PSA peptide	Montanide ISA-51	recurrent PCa after radical prostatectomy; Phase II pilot, NCT00109811	5, s.c.	No serious AE. No significant changes in serum PSA.	[94]/2009
PSA, PSCA, PSMA, Survivin, Prostein, TRP-P8	14-synthetic-multi-peptide vaccination cocktail	± (imiquimod, GM-CSF or mucin-1-mRNA/protamine complex) + montanide ISA51	HSPC; Phase I/II	19, s.c.	Well tolerated; no patient showed any severe AE. A clinical response was observed in 8 out of 19 patients and PSA-DT was improved in 4 cases.	[95]/ 2009
Ii-Key/HER-2/neu	AE37	GM-CSF	castrate-sensitive and CRPC; Phase I	32	Safe. AE37 elicited HER-2/neu-specific cellular immune responses.	[96]/ 2010
NY-ESO-1	NY-ESO-1 peptides	CpG 7909	advanced PCa; Phase I	13	Induced integrated antigen-specific antibody immune responses; T cell responses were induced in 9 patients (69%).	[97]/ 2011
SART3, MRP3, ppMAPkkk, HNRPL, EGF-R, PSMA, UBE2V, p56^lck^, CypB, PAP, SART2, PSA, WHSC2, EZH2, PTHrP	PPV (2-4 selected peptides)	Montanide ISA51V	CRPC; Phase II	100	PPV was safe and well tolerated. Peptide-specific IgG and T cell responses strongly correlated with PSADT, and with OS.	[98]/ 2013
hTERT	GX301 (4 telomerase peptides)	Montanide ISA-51, Imiquimod	PCa; Phase I/II	11, i.d.	Safe, well tolerated. With potential immunologic and clinical efficacy, vaccine-specific immunological responses were detected in all patients.	[99]/ 2013
NY-ESO-1	NY-ESO-1 peptides	/	mCRPC; Phase I	9, s.c.	NY-ESO-1 specific T cell response in 6 P; PSA DT increased from 3.1 to 4.9 months.	[100]/ 2014
SART3, Lck, UBE2V, WHSC2, HNRPL, MRP3, PAP, PSMA, PSA, EGF-R, PTH-rP, CypB	KRM-20 (mixture of 20 peptides)	Montanide ISA51V	CRPC; Phase I UMIN000008209	17	Safe; no serious AE. Partial response or no change in PSA observed in 7/15 patients (47%); CTL activity for at least one peptide and IgG level were augmented in most patients.	[101]/ 2015
hTERT	UV1 long peptides	+ GM-CSF	mPC; Phase I/IIa	21, i.d.	Moderate toxicity; UV1-specific T cell responses in 18/21 patients (85.7%).	[102]/ 2017
CDCA1 (cell division cycle-associated 1)	CDCA1 peptide	Montanide ISA51	CRPC post-DBC; Phase I NCT01225471	12, s.c.	Well tolerated without any serious AE; Peptide-specific CTL responses.	[103]/ 2017
RhoC	synthetic long peptide of RhoC	Montanide ISA-51	PCa with radical prostatectomy; Phase I/II	22	Well tolerated; a strong CD4 T cell response.	[104]/ 2020
hTERT	GX301 (4 telomerase peptides)	Montanide ISA-51, Imiquimod	mCRPC; Phase II 2014-000095-26; NCT02293707	63, i.d.	No major side effects, 54% overall immune responder rate, 95% of patients showed at least one vaccine-specific immune response.	[105]/ 2021
**Tumor Cell-Based Vaccines**						
**Cells**		**Stimulatory Adjuvants**	**Target; Phase**	***n*** **, Appl. Route**	**Outcomes**	**Ref/** **Year**
Autologous, irradiated tumor cells engineered to secrete GM-CSF		GM-CSF	PCa; Phase I	8	Well tolerated. Induction of anticancer immunity as assessed using DTH skin testing; new antiprostate cancer cell antibodies were detected.	[106]/ 1999
Three tumor cell lines + Mycobacterium vaccae (SRL-172)			CRPC; Phase I/II	60	Safe and well tolerated with no major AE. No significant decrease in PSA, an increase in cytokine production, increases in specific antibodies and evidence of T cell proliferation in response to the vaccinations.	[107]/ 2002
Three allogeneic cell lines + bacille Calmette-Guérin			CRPC; Phase I	28 i.d.	No significant toxicity. In total, 11/26 patients (42%) showed significant, prolonged decreases in PSA velocity.	[108]/ 2005
LNCaP and PC-3 irradiated and engineered to secrete GM-CSF (GVAX plat-form)		GM-CSF	PCa with PSA relapse + radical prostatectomy; Phase I/II	21	Favorable safety profile. Significant decrease in PSA velocity.	[109]/ 2006
			mPCa; Phase I/II	80, i.d.	Well tolerated, no serious AE. PSA stabilization occurred in 15 (19%) patients, and a >50% decline in PSA was seen in 1 patient.	[48]/ 2008
Autologous tumor cells, irradiated		Immunomodulated with IFN-α2b and BCG	mPCa; Phase I	11	Safe; AE restricted to the inoculation sites. Two patients had a decrease in PSA.	[110]/ 2007
LNCaP irradiated and engineered to express recombinant IL-2 and IFN-gamma		IL-2 and IFN-gamma	CRPC; Phase I	6	Safe and feasible. PSA decline of 50% was achieved in two of the six patients.	[111]/ 2007
			CRPC; Phase I/II	30, i.d.	Safe and well tolerated. Significant prolongation of the PSA-DT, 3 patients sustained a >50% decrease in PSA, T cell stimulation in the majority of patients.	[112]/ 2009
Two allogeneic prostate tumor cell lines irradiated and engineered to express αGal epitopes		HAP (HyperAcute Prostate)	advanced PCa; Phase I	8	Minimal toxicity. Humoral immune responses to autoantigens in 25% of P (2/8), suggesting dose-dependent effect.	[113]/ 2013
**Dendritic Cell-Based Vaccines**						
**TA**	**Cells**	**Stimulatory Adjuvants**	**Target; Phase**	***n*** **, Appl. Route**	**Outcomes**	**Ref/** **Year**
Loaded with PSMA peptides: PSM-P1 or PSM-P2	aDC		CRPC; Phase I, Phase II	19 and 33, i.v.	No significant toxicity. Increased T cell response to PSMA peptides in HLA-A2-positive patients; 7/19 and 9/33 partial PSA value responders.	[114]/ 1996 [115]/ 1998
hrPSA	aDC		PCa after radical prostatectomy; Phase I	24, i.v., s.c., i.d.	No serious AE. Transient PSA decrease; disappearance of circulating prostate cells.	[116]/ 2004
Loaded with hTERT I540 peptide	aDC		CRPC; Phase I	5	No significant toxicity. hTERT-specific T lymphocytes were induced in 2 patients.	[117]/ 2004
Loaded with allogeneic prostate cancer cell line lysate (LNCaP, DU14, JM-RCC)	aDC	KLH	CRPC; Phase I/II	11, i.n. or i.d.l	Feasible and not toxic, induction of both humoral and cellular immunity, a reduction in PSA velocity in 1 and an increased PSA-DT in 6 men.	[118]/ 2004
Loaded with PAP + GM-CSF (sipuleucel-T)	aDC		mCRPC; Phase III multicenter NCT00065442	82 and 341, i.v.	Well tolerated. Beneficial treatment effect: increased specific T cell response. TTP and interim survival were associated with a subset of subjects with Gleason scores ≤ 7; prolonged OS for 4.1 months.	[119]/ 2005 [10]/ 2010
Loaded with a cocktail peptide PSA, PSMA, survivin, prostein, trp-p8	DCs		CRPC; Phase I	8	Safe and feasible; no serious AE. One partial response in PSA (decrease >50%) and three stable PSA values or decelerated PSA increases. Three of four PSA responders also showed antigen-specific CD8+ T cell activation against prostein, survivin and PSMA.	[120]/ 2006
Loaded with PSA peptide (PSA146-154)	aDC		locally advanced or mPCa; Phase Ib	14, i.v.	DTH-derived T cells exhibited PSA peptide-specific cytolytic activity.	[121]/ 2006
Loaded with peptides derived from PSCA, PAP, PSMA, PSA	aDC		CRPC; Phase I/II	6, i.d.	Well tolerated. Significant cytotoxic T cell responses against all prostate-specific antigens tested; an increase in PSA-DT.	[122]/ 2006
Loaded with PSCA and PSA peptides	aDC		mCRPC; Phase I/II	12, s.c.	No relevant toxicities. DTH positivity was associated with significantly superior survival.	[123]/ 2006
Loaded with PSA peptides (PSA-1, PSA-2, PSA-3)	aDC	IFN-gamma	mCRPC; pilot	12, i.c.	Well tolerated; no serious AE. In total, 2/12 had slight increase in PSA peptide-specific T lymphocytes; 1 partial and 1 mixed responder were identified.	[124]/ 2007
Loaded with a peptide cocktail: PSA, PAP, PSMA	aCD1c	KLH	mCRPC; Phase I	12, i.d. or i.v.	Feasible, safe and well tolerated.	[125]/ 2008
Loaded with apoptotic LNCaP tumor	aDC	KLH	CRPC; Phase I	12, s.c.	Safe and well tolerated. Increase in T cell proliferation responses to prostate tumor cells in vitro, decrease in PSA slope, two-fold increase in PSA-DT.	[126]/ 2010
Loaded with prostate cancer cell line lysates (DU145, LNCaP, PC3)	alogeneic DC	CCH, TRIMEL	CRPC; Phase I	14, s.c.	Safe; no relevant AE. In total, 6/14 had decrease in PSA levels; DTH(+) patients showed a prolonged PSA-DT.	[127]/ 2013
Loaded (incubated) with rPSMA, rSurvivin peptides	DC		CRPC; Phase I	11, s.c.	Cellular immune response, disease stabilization, no adverse events and partial remission.	[128]/ 2015
Tn-MUC1 loaded	aDC		nmCRPC; Phase I/II	17, i.d., i.n.	Safe, able to induce significant T cell responses and increase in PSADT following vaccination.	[129]/ 2016
Loaded with protein PA001—contains the extracellular domain of hPSMA	aDC	Transduced with Ad5f35-encoding inducible human (ih)-CD40	mCRPC; Phase I	18, i.d.	Safe. Anti-tumor activity was observed with PSA declines; objective tumor regressions and robust efficacy of post-trial therapy.	[130]/ 2017
Loaded with irradiated prostate cancer cell line LNCaP (DCVAC/PCa)	aDC	Cyclophosphamide, Imiquimod	PCa with BCR; Phase I/II 2009-017259-91	27 s.c.	No significant side effects, PSA-DT in all treated patients increased after 12 doses from 5.67 months to 18.85 months, specific PSA-reacting T lymphocytes were increased significantly.	[131]/ 2018
Incubated with NY-ESO-1, MAGE-C2 and MUC1	a-mDC + a-pDC	/	CRPC; Phase IIa NCT02692976	21	Feasible and safe. Induced functional antigen-specific T cells, which correlated with an improved clinical outcome.	[132]/ 2019
Electrofused with autologous prostate tumor cells (aHyC)	aDC	Cyclophosphamide, allogeneic buffy coat	CRPC; Phase I	19, s.c.	Safe, no serious AE and feasible. mOS was 58.8 months. Attenuates an increase in peripheral blood CD56^bright^CD16^−^ NK cells. A decrease in CD56^bright^CD16^−^ NK cells correlates with prolonged patient survival.	[11]/ 2021 [12]/ 2022
Loaded with mRNA from autologous TC or mRNAs that encoded hTERT and survivin	aDC	/	PCa patients after prostatectomy; Phase I/II	20	Safe; no serious AE. In total, 11/20 P were BCR-free over 96 months.	[133]/ 2022
**Mixed Cancer Treatment Vaccines**						
**TA**	**IT-Treatment Modality**	**Adjuvants**	**Target; Phase**	**N, Appl. Route**	**Outcomes**	**Ref/** **Year**
PSMA	DNA/Ad expression vector	±CD86 plasmid, ±GM-CSF	PCa; Phase I/II clinical trial	26, i.d.	No serious AE. In total, 100% of P inoculated with the viral vector and 50% of P receiving DNA plasmid showed signs of successful immunization.	[134]/ 2000
PRAME, PSMA	DNA plasmid + 2 peptides	/	PCa; Phase I	10, i.n.	Safe, feasible, well tolerated. In total, 4 of 10 P had stable disease (SD) for 6 months or longer, or PSA decline.	[135]/ 2011
PAP	sipuleucel-T ± pTVG-HP DNA	±GM-CSF	mCRPC; Phase I, pilot NCT01706458	18, i.v., i.d.	No AE > grade 2 were observed. Th1-biased PAP-specific T cell responses were detected in 11/18; higher titer antibody responses to PAP detectable in booster arm. The mOS was 28 months.	[136]/ 2018
hTERT (V934/V935)	Ad6expression vector ± DNA	/	PCa; Phase I, pilot NCT00753415	14, EP	Good safety profile, with no severe AE. Significant increase in immunogenicity response against hTERT.	[137]/ 2020

aDC, autologous DC; AE, adverse events; ART, ADP-ribosyltransferase; BCR, biochemical recurrence; CCH, *Concholepas concholepas* haemocyanin; CSPC, castration-sensitive prostate cancer; CTL, cytotoxic T lymphocytes; CypB, cyclophilin B; DBC, docetaxel-based chemotherapy; d.s., dermal scarification; EGF-R, epidermal growth factor receptor; EP, electroporation; HER-2/neu, human epidermal growth factor receptor 2; HNRPL, heterogeneous nuclear ribonucleoprotein L; HSPC, hormone-sensitive prostate carcinoma; i.c., intracutaneously; i.d., intradermally; i.m., intramuscular injection; i.n., intranodally; i.t., intratumoral; i.v., intravenous infusion; KLH, keyhole limpet hemocyanin; Lck, lymphocyte-specific protein tyrosine kinase; mOS, median overall survival; MRP, multidrug resistance-associated protein; *n*, number of patients; NY-ESO-1, New York esophageal squamous cell carcinoma 1 antigen; P, patients; PPFS, PSA progression-free survival; PSCA, prostate stem cell antigen; PPV, personalised peptide vaccine; PRAME, preferentially expressed antigen in melanoma; PSA-DT, PSA-doubling time; rF, recombinant fowlpox virus; rPFS, radiographic progression-free survival; rV, recombinant vaccinia virus; SART, squamous cell carcinoma antigen recognized by T cell 2; s.c., subcutaneously; STEAP1, six transmembrane epithelial antigens of prostate 1; TARP, T cell receptor gamma alternate reading frame protein; TRIMEL, standardized melanoma lysate; TRP-P8, transient receptor potential p8; TTP, time to progression; UBE2V, ubiquitin-conjugating enzyme E2 V; VRP, vaccine replicon particles; WHSC2, Wolf–Hirschhorn syndrome candidate 2 protein. Grey boxes: locally approved treatments.

##### Safety of DC-Based Vaccines

CRPC mainly affects older men who are compromised because of other accompanying diseases. They are usually receiving a range of other therapies and are consequently more prone to various treatment complications [138]. In individual cases, the therapy must be changed due to adverse effects (AEs) of drugs, known interactions with other drugs, accompanying diseases or patient wishes.

The safety of DC immunotherapy has been documented in several phase 1 clinical trials [139]. Local injection site reactions (e.g., pain, erythema and pruritus) were common but generally mild. Systemic AEs with fever, malaise and other flu-like symptoms were observed; however, grade 3–4 systemic AEs according to Common Terminology Criteria for Adverse Events (CTCAE) were extremely rare [140]. It is possible to trigger autoimmune reactions with all types of immunotherapy. DC-based cancer treatment vaccines have been shown to rarely cause severe AEs, in contrast to other immunotherapeutic approaches such as monoclonal antibodies and cytokines [141]. In one study, up to 60% of patients treated with ipilimumab had AEs due to immune reactions (of which 15% were CTCAE grade 3–4) [142]. In contrast, patients treated with DC maintained their quality of life because of the low incidence of AEs [143]. Quality of life is an important indicator that we use for evaluating new cancer drugs. Reports on the impact of DC immunotherapy on quality of life are rare. One study evaluating 55 patients with renal cell carcinoma treated with DCs showed no negative effect of immunotherapy on quality of life [143].

Accordingly, the results of a recently completed clinical trial, in which the cell vaccine was prepared from DC and tumor cells, show a relatively high level of safety of treatment with aHyC [11]. The results also showed that the patients maintained a high level of functionality, remained active and self-caring and had quality free time, which contributed to psychological relief and a reduction in mental distress due to the disease. It should be noted here that the small sample (*n* = 16) dictates caution in interpreting the results.

aHyC therapy exhibits a favorable safety profile that can be attributed to several factors. First, the aHyC cell vaccine is completely autologous; tumor cells and DCs as the starting material for the production of immunohybridomas are obtained from the patient’s tissue and no other starting materials and raw materials are present in the final product. Second, we did not administer aHyC intravenously, but subcutaneously. Intravenous administration is generally not used for vaccination because it elicits a relatively small immune response compared with other injection routes [144], and may also cause allergic reactions. We believe that due to the completely personalized preparation of aHyC, there were no allergic or autoimmune reactions. Moreover, the lower incidence and lower intensity of AEs with aHyC compared with sipuleucel-T (administered intravenously) could be due to the fully autologous nature of the vaccine, quality of preparation and subcutaneous administration. No patient required hospitalization, no autoimmune reactions were detected and laboratory indicators of liver and kidney functions remained stable during the clinical investigation, which indicates that immunotherapy with aHyC does not affect the functioning of important organs in the body.

## 3. Adoptive Cell Transfer

Adoptive cell transfer refers to a cell-based anticancer immunotherapy that involves several modalities of the collection, manipulation and re-administration of lymphocytes. Collected lymphocytes can be autologous or allogeneic and are either circulating or tumor-infiltrating leukocytes. CAR-T therapy, the most studied and already approved therapy for the treatment of some non-solid cancers, involves the collection of circulating T lymphocytes, which are then genetically engineered to express tumor antigen-specific receptors together with a costimulatory domain to activate T cells upon antigen recognition (chimeric antigen receptors (CARs). The chimeric molecule enables T cells to increase their responsiveness to an antigen by increasing their proliferation and cytokine secretion.

An important challenge with this therapy is identification of the tumor antigen, which should be present on most cancer cells, but not on normal cells. Another important challenge is how to reduce the toxicity of such therapy, which is mostly connected to the serious side effect known as cytokine release syndrome, an increase in inflammatory cytokines released by immune cells after CAR-T transfer, but also due to neurotoxicity as a result of targeted antigens on normal cells by CAR-T cells, so-called on-target/off-tumor recognition [145]. The third important challenge with CAR-T treatment is the durability of the therapy. CAR-T is considered a passive form of immunotherapy because it does not (re)activate the immune system but has an intrinsic antineoplastic effect. In contrast, DC-based vaccines are examples of active immunotherapy, because they achieve anticancer effects only upon activation of the host’s immune system [146]. Thus, patients who respond to CAR-T therapies are at risk of disease recurrence due to several factors, including the lack of long-term persistence after CAR-T transfer [147]. However, various strategies are currently being tested to improve the long-term efficacy of CAR-T treatment, including consolidative treatments, patient selection, additional pre- and post-CAR-T infusion treatment and CAR-T design and manufacturing optimization [148].

These challenges, together with the immunosuppressive tumor microenvironment and the physical barriers of solid tumors, are the reason that the use of CAR-T therapy is currently limited mainly to blood cancers, but despite all the difficulties, many clinical and preclinical studies are currently underway for solid tumors, including PCa. Three antigen candidates meet the criteria for CAR-T therapy for PCa and are currently under investigation: the epithelial cell adhesion molecule, prostate stem cell antigen and PSMA (reviewed by Perera et al. [149]).

## 4. Limitations of Immunotherapy in Prostate Cancer

### 4.1. Tumor Microenvironment

PCa tissue is composed of tumor cells and host components (immune cells, stroma, epithelial cells and soluble factors (cytokines)) that form the tumor microenvironment (TME). TME changes with time. There are constant interactions between immune cells, stromal cells, non-cellular components and tumor cells that affect tumor progression/regression or the response to treatment [16]. Both tumor cells and tumor stroma create an unfavorable environment for an effective immune response, leading to tumor progression and escape [150]. The stroma contains fibroblasts, which allow tumor cells to survive, and in addition, they participate in the development of a hypoxic environment, which is extremely unfavorable for the normal functioning of immune cells [151]. In such conditions, immunosuppressive cell populations are recruited, and the function of antitumor effector CD8^+^ T lymphocytes and DCs is inhibited [152,153]. Research into the functioning of the immune system in and around PCa tissue has confirmed the presence of regulatory T lymphocytes (Treg), M2 tumor macrophages and myeloid-derived suppressor cells. In addition, many other suppressor mechanisms have been discovered that favor the survival of cancer cells and participate in the spread of cancer, such as cytokines secreted by fibroblasts, tumor cells and stromal cells, as well as adenosine [154,155]. PCa is classified as one of the “immune cold cancers” [156]. Relatively few T lymphocytes were measured in TME. This is partly due to the low total tumor mutational burden (TMB) observed in PCa compared with other cancers (e.g., malignant melanoma, renal cancer), resulting in a reduced presence of tumor neoantigens, which are required for an effective immune response [157,158]. In addition, tumor cells evade the immune system by changing their surface antigens, so that the immune system no longer recognizes them as foreign, and therefore the disease progresses more easily. The key to the successful treatment of CRPC with new immunotherapeutic approaches is understanding the complexity of tumor cells and their interactions with the highly immunosuppressive TME of PCa [159] and thus to understand the different mechanisms of cancer cell resistance to evade immune surveillance. Contact-dependent and paracrine communication between tumor cells and host cells, extracellular matrix remodeling by cancer cells, properties of tissue-resident immune cells, altered metabolic demands and metabolite secretion in TME [160] and numerous other factors tailor the unique features of each TME, which dictates a unique approach to treat cancer in each individual.

### 4.2. Biomarkers

Most clinical studies to date have not shown a benefit of DC-based immunotherapy (e.g., effect on PSA values, time to disease progression, symptoms), most likely due to inadequate surrogate targets that did not correlate with prolonged survival [55,161]. Although pioneering in nature, the IMPACT study has been subject to many criticisms. Patients receiving sipuleucel-T received docetaxel as next-line treatment in most cases at disease progression, whereas patients receiving the placebo received sipuleucel-T at disease progression. This has led to a delay in treatment with drugs known to be effective [162]. In addition, the therapy did not significantly affect certain study endpoints: the time to disease progression, significant effect on PSA, tumor burden, symptoms or pain [10]. Thus, without a significant impact on surrogate endpoints, it is difficult to understand and explain the observed improvement in survival of patients treated with sipuleucel-T. Moreover, no biological marker was found in the patients’ blood that could be used to evaluate the effectiveness of the vaccine.

Without a known predictive biomarker to assess the effectiveness of therapy, it is difficult to guide treatment and assess a disease status and progression. It is believed that the kinetics of the clinical response after immunotherapy with antitumor vaccines are different (delayed, prolonged) compared with cytotoxic therapy or therapy with second-generation antiandrogens [163]. In the field of PCa, many biomarkers have been considered for prognostic assessment or treatment decisions, but only a few have actually been rigorously tested and validated [164,165]. One such biomarker is a constitutively expressed splice variant of the androgen receptor AR-V7. The variant AR-V7^+^ shows resistance to both enzalutamide and abiraterone acetate [166]. However, the presence of the AR-V7^+^ variant does not appear to impair the response to taxanes (e.g., docetaxel) [167]. On the other hand, there is a lack of biomarkers in the field of immunotherapy of PCa. It was found that patients with a high microsatellite instability (MSI-H) or mismatch repair deficiency (MMRD) respond better to ICIs [168,169]. Patients carrying germline mutations in DNA damage repair genes have also responded well to ICIs [170,171,172]. To date, the biomarker with likely the greatest potential clinical usefulness is the status of microsatellite instability [170,173,174], which has been particularly supported by the FDA with the approval of pembrolizumab for therapy of patients with mCRPC with MSI-H [175].

The expression level of programmed cell death protein 1 (PD-1) and programmed death-ligand 1 (PD-L1) is low in healthy prostate tissue (present in 0.5–1.5% of cases), but relatively high in PCa (7.7–13.2%) [176]. It is associated with the aggressiveness of the disease; PD-L1 expression is present in 61.7% of local high-risk PCa and 50% of CRPC [177,178]. Nevertheless, the response to ICI therapy does not necessarily depend on PD-L1 expression. This suggests that other factors contribute to the efficacy of anti-PD-L1 therapy and that simply measuring tissue PD-L1 levels may not be an effective biomarker of the treatment response.

According to currently available data, prognostic and predictive blood biomarkers in advanced PCa are not yet ready for use in daily clinical practice. Other markers are at various stages of development and evaluation. In the future, it will be imperative to discover alternative biological markers that will allow us to evaluate the response of the disease to immunotherapy and adjust the treatment in time, if necessary. Open challenges remain in identifying patients in whom conventional methods for assessing the response fail to identify a benefit from immunotherapy treatment. The key purpose of immuno-oncology-specific criteria for evaluating the response to therapy is to sensibly correlate and capture atypical response patterns, so that patients do not discontinue effective treatment prematurely or remain on ineffective treatment too long, and to identify patients who will respond to therapy. It is necessary to find reliable and properly validated biomarkers that can identify patients who will respond to therapy. The appropriate selection of patients who will respond to specific treatment will play an important role in the individual therapeutic approach. Predictive biomarkers (e.g., MSI-H, MMRD, TMB) have already been identified in some groups of patients who responded well to immunotherapy [169,179,180]. In addition, with a better understanding of the TME of PCa, we will be able to turn the so-called immune cold cancer into a hotter one.

Therefore, the changes in the NK cell subpopulation, which were discovered in the recently completed phase 1/2 study that linked their role to survival, show a perspective in the field of biomonitoring of CRPC disease [11,12]. Namely, it was found that immunotherapy with aHyC inhibits the growth of a subpopulation of NK cells (CD56^bright^CD16^−^) [11], and concurrently, among patients who died, survival was significantly longer in those with a smaller increase in this subpopulation of NK cells in peripheral blood. The relatively low number of patients included in this correlation analysis (*n* = 10) dictates caution in interpretation of the results; however, the correlation between the parameters, the CD56^bright^CD16^−^ change and survival, was high (*R* = −0.80; *p* < 0.005), suggesting that changes in the peripheral blood CD56^bright^CD16^−^ NK cell subpopulation could be a novel biomarker that could be helpful in evaluating the response to aHyC therapy. The role of these cells in the immune system is not yet clear. It has been reported that their number is high in the endometrium of the uterus, and this has been linked to a possible immunosuppressive role and protection of the fetus from rejection [181]. Also, in one study on multiple sclerosis treatment, their expansion was associated with a reduction in inflammation [182]. They act in an immunoregulatory manner, secrete cytokines and play a role in metastases [183,184], suggesting that immunohybridomas can interact with both T cells and NK cells in the immune response [55]. Data in this area are scarce [185,186], but warrant further studies.

## 5. Discussion

This article summarizes the various immunotherapy modalities for prostate cancer and emphasizes the potential of DC-based immunohybridoma cancer vaccines. PCa cells divide relatively slowly, even in patients with advanced disease, allowing time to activate the immune system [187]. This makes PCa an ideal target for cancer treatment vaccines [49]. PCa vaccines that work by activating the immune system may be more beneficial if used early, before the onset of high-burden disease [57]. There are several reasons for this: the immune system is activated with a delay, a certain time after administration of the vaccine; a greater disease burden reduces the effectiveness of the immune response; and cancer develops mechanisms by which tumor cells escape the control of the immune system.

PCa cells express several known prostate-specific immunogenic antigens (e.g., PAP, PSA and PSMA) and can be used as targets for vaccines based solely on one antigen [57,188]. Although vaccine-based therapies have several advantages, one possible drawback is that an effective immune response to a specific TAA might be variable, limited in part by human MHC, termed the human leukocyte antigen expression and haplotype, which affects the presentation of the immunogenic epitopes [189,190]. A high affinity and increased duration of peptide-MHC interactions may lead to more effective vaccine-induced immunogenicity [191,192].

Ways of presenting cancer cell antigens to the immune system have been investigated in various clinical studies, from a general strategy with one known antigen for all patients (easier preparation of the vaccine) to other, more individualized strategies with several different antigens for individual patients (more demanding vaccine preparation). A general approach was used in the PROSTVAC-VF vaccine trial (viral vector-based vaccine) [40,193] and the GVAX trial (tumor cell-based vaccine) [47,193]. Beneficial effects on patient survival were reported in some PROSTVAC-VF and GVAX studies in which the vaccine was administered subcutaneously/intracutaneously [193]. DNA-based vaccines containing information on various TAAs (PAP, PSA, PSMA and testicular cancer antigen) have not demonstrated an increased clinical efficacy, but most trials have shown an immunological response [33]. The strategy of using a single antigen, specifically PAP, was used in the production of sipuleucel-T in the IMPACT trial. Because most PCa cells express PAP [57], this was a logical next route to improve the efficacy of vaccines in PCa immunotherapy. The likely mechanism of action of the sipuleucel-T cell vaccine is the induction of an immune response to PAP on PCa cells via DCs [59]; however, it is not entirely clear whether sipuleucel-T actually works this way because it contains less than 20% DC markers [194,195]. DCs are capable of activating both naive and memory T lymphocytes and appear to be an ideal target for amplifying antitumor immune responses [53] in the treatment of patients with CRPC. Thus, an approach with enriched, activated DCs may be a more effective immunotherapy strategy than the approaches mentioned previously [132].

An innovative way to trigger specific immune reactions to interrupt the unresponsiveness of antitumor effector lymphocytes and NK cells, similar to those of spontaneous tumor rejection, was performed with an optimally planned and manufactured immune vaccine aHyC [63,64,65] in a phase 1/2 study, in which whole autologous tumor cells were used as a source of TAs, with known and unknown antigens specific to each patient [11], instead of choosing one [10] or a few [132]. In addition, all the patients included in the study had been pre-treated with metronomic doses of cyclophosphamide before the first vaccination, aiming to reduce the number of Treg cells in the TME [196,197,198].

An improved functional efficiency of aHyC, reflected in a better clinical picture of patients, could be achieved by optimizing the procedures for the preparation of the cell vaccine and by establishing the optimal functioning of the patient’s immune system, especially in the environment of a malignant neoplasm. Most tumors develop in immunocompetent hosts, which means that the progression of the disease from a localized form to metastatic disease is associated with numerous interactions of cancer cells with cells of the host’s immune system. Research into the functioning of the immune system in and around PCa tissue has confirmed the presence of Treg cells and many other suppressor mechanisms that favor the survival of cancer cells and participate in the spread of cancer. Thus, the goal of immune therapy in the future, with an optimally designed and manufactured immune vaccine, is to trigger specific immune reactions similar to those during spontaneous tumor rejection [199].

## 6. Conclusions and Future Directions

PCa has immunogenic potential, but the immunosuppressive TME prevents the immune system from responding appropriately to destroy tumor cells. Populations of inhibitory immune cells, fibroblasts and tumor cells produce cytokines that indirectly and directly inhibit the immune system, which should fight against tumor cells. In recent years, immunotherapy has had a profound impact on the treatment of metastatic cancer and has changed the standard of care for many types of tumors. Although some disseminated cancers, such as malignant melanoma, lung cancer, bladder cancer and renal cell cancer, have shown dramatic responses to immunotherapy [7], PCa has generally not shown a significant response. In contrast to other cancers, when blocking the PD-1/PD-L1 axis alone is sufficient, it is likely that different approaches will need to be combined in PCa to improve clinical response rates. Results of ongoing phase 2 and 3 clinical trials may provide insights into the advantages and disadvantages of different strategies, as well as into different mechanisms of therapeutic resistance.

Adequate platforms for vaccine development will also need to be developed. Usually, animal models are used for such research, but the immune system of rodents is very different from that of humans, and the transfer of the data obtained is often useless [200,201]. In the era of personalized medicine, we strive to adapt the treatment to each individual patient based on the characteristics of the tumor (and the patient). In the near future, molecular dissection of PCa is inevitable, but additional research work will be required before we have a definitive clinical usefulness of specific biomarkers [202].

We believe that immunotherapy in PCa is just the beginning of a long story. With further scientific work, we will be able to answer many questions, including the following. How can we make PCa more “hot”—prone to attacks by the immune system or immunotherapy? What is the exact mechanism by which an effective immune response is blocked? Are there different cancer cell-killing T cells that are activated by CTLA-4 inhibition versus those that are activated by PD-L1 inhibition? It is still difficult to predict what role aHyC immunotherapy will play in the future. The effect of aHyC on CD56^bright^CD16^−^ NK cells, suggesting an association with survival, is certainly an important finding. With further research, we may be able to confirm this and discover something new, opening up possibilities in the field of PCa and other forms of solid tumors. In the coming years, we will obtain a clear answer about the role of new forms of systemic treatment, which may be used in combination and at earlier stages of the disease.

## Data Availability

Not applicable.

## Abbreviations

AE, adverse effect; APC, antigen-presenting cell; AR, androgen receptor; ATMP, advanced therapy medicinal product; CAR, chimeric antigen receptors; CTCAE, Common Terminology Criteria for Adverse Events; CTLA-4, cytotoxic T lymphocyte-associated protein 4; CRPC, castration-resistant prostate cancer; DC, dendritic cell; FDA, US Food and Drug Administration; GM-CSF, granulocyte–macrophage colony-stimulating factor; hTERT, human telomerase reverse transcriptase; ICI, immune checkpoint inhibitor; MHC, major histocompatibility complex; mHSPC, metastatic hormone-sensitive prostate cancer; MMRD, mismatch repair deficiency; MSI-H, high microsatellite instability; NK, natural killer; PAP, prostatic acid phosphatase; PCa, prostate cancer; PD-1, programmed cell death protein 1; PD-L1, programmed death-ligand 1; PSA, prostate-specific antigen; PSMA, prostate-specific membrane antigen; TA, tumor antigen; TAA, tumor-associated antigen; TMB, tumor mutational burden; TME, tumor microenvironment; Treg, regulatory T lymphocyte; TSA, tumor-specific antigen.
